# ‘Well, if the kids can do it, I can do it’: older rehabilitation patients' experiences of telerehabilitation

**DOI:** 10.1111/hex.12443

**Published:** 2016-02-18

**Authors:** Wendy Shulver, Maggie Killington, Claire Morris, Maria Crotty

**Affiliations:** ^1^ Rehabilitation, Aged and Extended CareFlinders UniversityDaw ParkSAAustralia; ^2^ Rehabilitation and Aged CareRepatriation General HospitalDaw ParkSAAustralia

**Keywords:** home rehabilitation, older people, Telehealth, telerehabilitation

## Abstract

**Background and objective:**

Although trials continue to emerge supporting the role of telerehabilitation, implementation has been slow. Key users include older people living with disabilities who are frequent users of hospital rehabilitation services but whose voices are rarely heard. It is unclear whether the use of technologies and reduced face‐to‐face contact is acceptable to these people. We report on a qualitative study of community dwelling participants who had received a home telerehabilitation programme as an alternative to conventional rehabilitation.

**Design:**

Thirteen older participants, three spouses and one carer were interviewed. All had participated in an individualized therapy programme, using a combination of face‐to‐face and video consults with therapists. The programme used ‘off‐the‐shelf’ technologies including iPads for videoconferencing and electronic FitBit^R^ devices. Interviews were recorded, transcribed verbatim and analysed using NVivo software.

**Results:**

Thematic analysis resulted in five emergent themes: (i) telerehabilitation is convenient; (ii) telerehabilitation promotes motivation and self‐awareness; (iii) telerehabilitation fosters positive therapeutic relationships; (iv) mastering technologies used by younger relatives is a valued aspect of telerehabilitation; and (v) Telerehabilitation does not replace traditional face‐to‐face rehabilitation therapies.

**Conclusions:**

Participants found telerehabilitation convenient and motivating, coped well with the technology and developed positive therapeutic relationships. The learning and practice aspects sat well in the context of a rehabilitation programme. The use of commercially available technologies may have contributed to respondents' high levels of acceptability. The perception of telerehabilitation as complementary to in‐person care and the expectation of technological support have implications for the implementation and delivery of telerehabilitation services to older people.

## Introduction

Telehealth technologies have been promoted as a solution to the challenges created by an ageing population with long‐term complex healthcare needs, by enabling provision of cost‐effective, quality and flexible health and social care.^1^, ^2^ In Australia, the Productivity Commission Inquiry Report *Caring for Older Australians* acknowledges that ‘fundamental reform is required’ to respond to current and future challenges that exist in Australia's aged care system. These challenges include a significant increase in the number of older people, an increasing incidence of age‐related disability and disease and rising expectations about the type and flexibility of care that is received. Proposed reforms include the development of ‘new, cost‐effective assistive and information technologies that offer opportunities for productivity gains and higher quality care’ and the choice, where appropriate, for older people to receive care at home.^3^

Telehealth involves the ‘remote exchange of data between a patient and healthcare professionals as part of the patient's diagnosis and healthcare management’.^1^ Telecommunication technologies enable transfer of information in the form of voice, data and images between patients and healthcare providers. Some examples include remote monitoring of blood pressure, blood glucose or activity levels (using an electronic pedometer device), or consultations conducted via videoconference from the patient's home, instead of travelling to an appointment. Telehealth in the home interventions often target older people as they offer promise for improving quality of life, more independent living and providing cost‐effective services.^4^

Whilst telehealth interventions have been shown to improve clinical indicators, successful implementation and adoption of telehealth has been slow and fraught with failure.^1^, ^4^, ^5^ Evidence from efficacy trials is not sufficient to guarantee successful implementation and adoption of new models of care and, if not context specific, may not be able to predict uptake and outcomes of an intervention ‘in a complex, dynamic context such as home care for older people’.^6^, ^7^, ^8^

Telerehabilitation is defined as the delivery of rehabilitation services using telecommunications technology.^9^ As for telehealth applications overall, the success of telerehabilitation interventions has been demonstrated by efficacy trials.^10^, ^11^, ^12^ However, it is unclear whether the use of technologies and reduced face‐to‐face contact with therapists is acceptable, particularly to older rehabilitation patients, due to the paucity of studies examining patient viewpoints in the context of telerehabilitation.^9^ A systematic review of telerehabilitation research found a high level of patient satisfaction with telerehabilitation.^12^ Crotty *et al*.^13^ investigated the feasibility of providing home‐based rehabilitation to older people using ‘off‐the‐shelf’ technologies and found that patients and clinicians were generally positive about this form of service delivery, whilst gains could be made in access, frequency and intensity of therapy. There are, however, very few in‐depth explorations of patient experiences with home telerehabilitation programmes, with most patient‐centred studies focussing on patient satisfaction using quantitative surveys.^9^ Of three recent qualitative studies identified in the literature, one explored the viewpoints of patients who had not experienced telerehabilitation.^14^ All three examined telerehabilitation in the context of a specific condition: chronic pain, total knee arthroplasty and shoulder joint replacement.^9^, ^14^, ^15^ This study addresses the paucity of qualitative literature examining patient experiences of home‐based telerehabilitation programmes. We aimed to address the following research questions: (i) How do community dwelling older people experience rehabilitation programmes using telehealth technologies? and (ii) How acceptable are telehealth technologies to older people in the context of rehabilitation?

## Methods

The study was nested within a larger evaluation of telehealth in the home being conducted from the Repatriation General Hospital, South Australia. Participants were provided with ‘off‐the‐shelf’ technologies including an iPad equipped with videoconferencing technology, as well as a FitBit^R^ activity monitor. Participants were visited by a physiotherapist and shown how to use the technology. Goals were developed each week, and patients were provided with an 8‐week individualized therapy programme including a range of exercises. Each week of the intervention period, patients received a visit at home and an additional videoconference via iPad from therapists. Activity data from the FitBit^R^ (i.e. number of steps taken per day) were visible to both therapist and patient via the iPad and discussed during video consultations. The study was conducted in a peri‐urban area some 50 km from the city of Adelaide and 40 km from the Repatriation General Hospital. All participants who had at the time completed the telehealth in the home programme were given the opportunity to participate in the qualitative study. Of a total of 15 participants who had completed the rehabilitation programme, 13 agreed to participate. The study was approved by the Southern Adelaide Clinical Human Research Ethics Committee.

Thirteen qualitative interviews were conducted with a total of 17 (six male, 11 female) participants. Of these, 13 were patients, three were spouses of patients, and one was a carer. Spouses and carer were interviewed jointly with the patient. Patient participants were aged between 60 and 92 years and were receiving treatment predominantly for problems with mobility.

The semi‐structured interviews were conducted in participants' homes and took between 0.5–1 h. Participants were asked about their experiences with the programme, prior experience with computer and iPad technology, usability of the technology, motivations for participation, challenges of receiving health care via telehealth, the quality of the care, their preferences between traditional and telehealth models of health care, and any other input they had regarding telehealth.

Interviews were recorded, transcribed verbatim and analysed using NVivo qualitative data analysis software by the researcher WS. Thematic analysis was undertaken to develop predominant themes that reflected participants' experiences of the telerehabilitation programme.^16^ An inductive approach to the thematic analysis was taken, with themes derived from the data itself, rather than related or linked to any pre‐identified theories or coding frameworks. As telerehabilitation experiences of older people are currently not well understood, this was an exploratory study, with the aim of providing descriptive insight rather than theorised analysis. A data driven approach enabled rich, unconstrained thematic description of participant experiences. A process of ‘topic coding’ served to organize the data. Derivative categories were created from each transcript, which were treated as a provisional framework for ‘analytic coding’ and ‘coding on’. These processes involve interpretive review of the material and the on‐going development of overarching themes and conceptual categories.^17^ Emerging themes were explored for the connections between them, drawing out patterns in the data and reflecting on their meanings. A number of strategies were employed to increase the authenticity of the findings. A sufficient number of interviews were conducted to achieve saturation. Transcripts were sent to participants for member verification, comment and clarification prior to analysis. Categories and themes were discussed and verified with a second researcher (MK) following both the initial and coding on phases.^18^ These verification processes included consideration of not only predominant themes, but also variations and exceptions, which have been included in the presentation of results.

## Results

Most participants reported positive outcomes in terms of the experience, activity levels, fitness, functioning and well‐being. Whilst a small minority did not feel that they obtained any benefit in terms of improving mobility, no participant was outright negative about the programme. Motivations for participation in the programme included the opportunity to get some exercise, social contact and learn how to use an iPad.

Patients' experiences of the telerehabilitation programme have been grouped into five emergent themes: (i) Telerehabilitation is convenient; (ii) Telerehabilitation promotes motivation and self‐awareness; (iii) Telerehabilitation fosters positive therapeutic relationships; (iv) Mastering technologies used by younger relatives is a valued aspect of telerehabilitation; and (v) Telerehabilitation does not replace traditional face‐to‐face rehabilitation therapies.

### Telerehabilitation is convenient

The time‐saving and convenience of not having to travel to appointments or exercise classes afforded by video consults was a consistent theme, especially for services located further distances away, such as in the city. The increase in convenience was perceived as less important in relation to the local GP (except when patients did not drive, accessing local services could still be problematic):

Because I don't drive at all, it's really difficult, transport‐wise, and it would've involved me in lots of time more than I needed to be spending doing that. [Patient 2]

[For] people who are rehabilitating after an operation and are away from the central areas, I think it's going to be a wonderful system. [Patient 5]

Physical discomfort and illness associated with travel could be a reason for preferring a video consult:

[Specialist is] the other side of Adelaide and just to get an appointment to go and talk to him, we felt, was ridiculous! [Patient is] not comfortable in a van and to drive all the way over to there just to have a little talk that could be over Skype – why not use Skype if we can? [Carer 7]

### Telerehabilitation promotes motivation and self‐awareness

Using telerehabilitation entailed reduced face‐to‐face visits. FitBit^R^ technology in combination with scheduled video consults motivated participants to keep up with their exercises/movement. No participant expressed a concern that the programme was invasive:

I found because I was doing [steps] that I was really conscious of it. So I was doing perhaps more steps than I normally would. And I found that if you have to go out for everything, you can get around 8,000 … The best things would be… they keep you in line. You don't slack off at all because you know that on the Monday, on the Wednesday or Friday, there's going to be someone there to talk to you. So you keep up with everything that you've been doing so you can give a report on those days. [Patient 1]

### Telerehabilitation fosters positive therapeutic relationships

Participants commented on the social contact the relationships with therapists and technical staff provided:

We just talked about anything and everything – what I've been doing that week, have I gone walking, riding, or whatever, and just had a general chat about anything… it was great. ‘Coz once you get to know the person at the other end … The physios were really warm and close and talked about anything and everything, not just what's wrong with you, but how you've been today and what you've been doing. [Patient 6]

Some participants felt telerehabilitation afforded therapists more time with them:

I found it easier to ask questions. Easier than when I go to a doctor. I usually come out without answers because I don't ask the right questions … I think it's probably the process of waiting in a waiting room for your turn and it's always later than it should've been and you go in there and you'd better hurry up because the next one's going to run late, too. I think a lot of that comes into it when you go to clinics and that sort of thing. [Patient 5]

Privacy and confidentiality were not identified as concerns, and there was generally a sense of trust that the therapist would protect patient privacy by ensuring the security of the video consult transmission:

[Privacy issues] didn't even cross my mind. I think she established trust personally … you do have a sense of trust. [Patient 2]

### Mastering technologies used by younger relatives is a valued aspect of telerehabilitation

Most patients had little or no first‐hand experience with computers and technology, and only one had previously used an iPad. Patients were generally positive about using the technology, with the small number who were initially apprehensive about it becoming quite comfortable, in some cases enthusiastic, with experience. Indeed, the opportunity to learn how to use technology usually associated with younger people (i.e. an iPad) was a factor in the decision for some participants to take part in the programme:

Well, if the kids can do it, I can do it. [Patient 1]

Well, I say to my grandchildren, when I was born, TV wasn't even invented! And you see all these things now. This wasn't invented, that wasn't invented, we didn't have this, we didn't have that, but you've got to embrace technology. [Patient 13]

One patient felt daunted at the prospect of using technology, and this was a factor in his original decision to decline participation:

[Before the programme] I wouldn't have had anything to do with an iPad … [wife] had a laptop and a computer down there but I never went near them … I just didn't want anything to do with it. The thing is that I've got no electronic or knowledge or anything like that … I thought I'd get through life without it. [Patient 7]

He eventually did take part, and he and two other participants purchased or were looking to purchase their own iPads after participating in the programme and had a growing awareness of what they can be used for (e.g. Skyping family, taking and storing photographs, accessing the internet).

There was however an expectation that the therapy team would provide technical support and respond rapidly to problems:

It's just that if something goes wrong, I don't know… As long as you've got phone numbers to ring, I think that would be the biggest thing to have and to let people know that the system's not working, what can I do? Or can somebody come and fix it? Or when? So you've got some peace of mind that you have done something about it, let somebody know about it. [Patient 4]

Whilst patients were generally enthusiastic about using the technology, some usability issues were highlighted and suggestions for improving the usability of the iPad, particularly for people with significant disabilities. These related to positioning of the iPad, provision of instructions and turning the iPad off and on:

The iPad was easy. The only trouble … the iPad was on a stand which looked up this way, so I'd have to sit somewhere and the person at the other end of the iPad had to see what they were doing. I tried it with a chair out there and the iPad on the floor – that's the only way they could really see [me demonstrate my exercises]. But if someone that's incapacitated or can't bend down or if you're older – 80 or 90 – you've got to find some way of putting that iPad so the person at the other end can see what you're doing. [Patient 6]

### Telerehabilitation does not replace traditional face‐to‐face rehabilitation therapies

Despite a generally positive reception, there was a strong view that video consults cannot entirely negate the need or desire for face‐to‐face consults. Although the convenience of telehealth was consistently appreciated, there were some instances when a face‐to‐face consult was preferred. Moreover, although telerehabilitation was not seen as detrimental to the therapeutic relationship, it was felt that the absence of the physical presence of the therapist limited what could be done at distance. This limitation encompassed the subthemes of physical examination, patient safety and intimacy:

Well, no, because one thing, if you're going through video link, they can't take your temperature or your pulse or anything else or really sort of check any symptoms. It's purely verbal. [Patient 3]

Although some participants were not concerned about safety, others thought that patient safety may be an issue with video consults:

If you're not one‐on‐one and they do something wrong with a dumbbell or something, hit themselves on the head, you've really got to get someone there in a hurry or make sure that someone else is in the house … When they're one‐on‐one, like a person here, they're doing exercise and the physio's right there with you, if you just stumble or whatever, they can grab you. [Patient 6]

Some participants also preferred the more personal nature of a face‐to‐face consult rather than a video consult, although others saw little or no difference between a face‐to‐face and video consult. One patient thought that videoconferences alone would be too isolating for a person largely confined to the home due to disability, and that some human contact is important. Another acknowledged that some people would be uncomfortable talking to a screen rather than in person.

Although the carer acknowledged that some of the limitations associated with ‘distance’ health care could be alleviated by having someone with the patient to support the videoconference (i.e. by helping with exercises under the guidance of the therapist), participants generally felt that it was important to intersperse face‐to‐face consults with video consults, in order to mitigate some of these concerns. Thus, the value of videoconferences was seen as supplementary to face‐to‐face consults:

I don't think I'd go too long on just videoconferencing. I think we've got to intersperse it somewhere along the line to be personal. [Patient 5]

But I think also, having met the person in person and then relating to them is the advantage … But certainly having contact with [therapist] and seeing her … in the early stages on a regular basis set the stage … with this iPad. [Spouse 7]

## Discussion

This study builds on the limited in‐depth literature examining patient viewpoints and experiences of telerehabilitation. The findings presented here align with previous literature, which reports that telerehabilitation approaches are acceptable to older rehabilitation patients.^12^, ^19^ In support of the authenticity of the results, the themes outlined in this study mirror many of those found by other qualitative studies investigating patient experiences with telerehabilitation programmes, including the convenience of not having to travel to appointments, supportive therapeutic relationships, a preference for telerehabilitation in combination with in‐person consults and usability of technology.^9^, ^14^, ^15^

Participants in this study were very positive about the programme and could see value in this model of service. In particular, participants described increased self‐efficacy as a response to the coaching approach provided by therapists through telerehabilitation. It has been argued that telehealth can both undermine individual agency or empower and foster independence, and that this can depend on the form the telehealth care takes.^8^ The participants in the present study did not experience telerehabilitation as disempowering. Instead, the programme fostered an awareness and interest in their activity levels, and in some cases also of new technologies. Video consult and activity monitoring were not seen as invasive, but rather appreciated and experienced as motivating. This study supports findings in a study by Eriksson *et al*.^15^ where participants reported a feeling of capability and independence on the telerehabilitation programme.

The current study suggests that provision of a FitBit^R^ device and distance monitoring of adherence to exercise through videoconferencing is acceptable and motivating for participants. Systematic reviews of randomized trials show that higher doses of exercise are associated with better outcomes in people after stroke and hip fracture, and greater falls prevention effects in older people.^20^, ^21^, ^22^, ^23^ The learning and practice aspects of this programme sat well in the context of a rehabilitation programme, suggesting that telerehabilitation is an acceptable way of encouraging increased activity levels and higher exercise uptake in older people.

Participants appreciated the convenience of not having to travel to appointments for rehabilitation services and recognized the value of telerehabilitation for people living in rural and remote areas, when travel is a significant barrier to accessing services. Yet most did not consider telerehabilitation to be an adequate substitute for traditional face‐to‐face models of service. In other words, participants saw telerehabilitation as complementary to face‐to‐face service delivery, rather than as an alternative. This could be a function of the way the telerehabilitation service was delivered. Beul *et al*.^24^ suggest that patients tend to prefer the model of service they have experienced. Telerehabilitation participants still received regular home visits from the therapist and also lived within reasonable distance of a rehabilitation facility, local hospital and their GP, thus were less limited in their service options than people living in more remote areas. Although it is persuasive to consider that had they received telehealth consults alone, participants' views may have changed, previous research contradicts this notion. Investigations of telerehabilitation programmes that have not interspersed video consults with face‐to‐face contact also expressed a preference for telerehabilitation in combination with face‐to‐face therapy, rather than a substitution for the latter.^9^, ^14^, ^15^

The results from these and the present study suggest that telerehabilitation is readily accepted by older patients in a supplementary capacity, but only as substitution to traditional models of service delivery when there is no other option. This has implications for the delivery of rehabilitation services, particularly to country and remote areas. Increasing access to healthcare services to rural and remote communities is a recognized benefit of telehealth.^25^ Research examining group‐based telehealth programmes indicates that people living in areas remote from healthcare services would also ideally prefer in‐person health care, but pragmatically recognize that options for this are limited and that telehealth has value as an alternative.^26^, ^27^ There is a paucity of research investigating telerehabilitation as a substitution to home visits for older people.^28^ Further research specifically investigating older patient experiences with home‐based telerehabilitation as a substitution to traditional therapy would give greater insight into the acceptability of telerehabilitation to remote areas where alternatives are limited, and possibly provide insights into the best method of delivery to increase acceptability.

Patients in this study were happy with the therapeutic relationship established with the therapists on the telerehabilitation programme; however, this may not have been as effective without the initial and intermittent face‐to‐face appointments which may have laid the foundation to the relationship. The regular social contact, easy communication and on‐going support were seen as positive aspects of the programme. In addition, participants were confident that the videoconference transmission was safe and their security was ensured. This mirrors the findings of Kairy *et al*. and Eriksson *et al*., yet contrasts those of Cranen *et al*. who reported that participants had concerns that the physical alienation from the therapist in telerehabilitation would be detrimental to the therapeutic relationship.^9^, ^14^, ^15^ However, the participants in Cranen *et al*.'s study had no first‐hand experience of telerehabilitation and were asked about it in a hypothetical sense. The contrast with patients experienced in telerehabilitation, including those in this study suggests that these fears may not be realized in practice.

Despite having little to no direct experience with iPad technology, the older people in this study were willing or even keen to give it a try. Initial apprehension, felt by some, was overcome with experience. Participants showed a willingness to experiment with iPad placement and room set‐up within their home to allow the coaching to occur efficiently. All were able to use the iPad for videoconferences. The use of ‘off‐the‐shelf’ technologies in this programme may have contributed to the high levels of acceptability among participants through seeing their children and grandchildren using such technology. This was cited by some as a motivating reason to enrol in the trial. Although older people use technology less than do younger people, particularly more recent technological innovations, little is known about older peoples' perceptions of modern touchscreen devices.^29^, ^30^ There is some evidence that the touchscreen interface of iPads and other tablets is acceptable and even preferred by older people, and that iPad use can have a positive impact on older peoples' social interaction and intergenerational communication.^4^, ^29^, ^31^ This study concurs with other research, which shows that older people can be interested in modern technology and successfully use it, particularly if it is perceived as useful.^4^, ^29^, ^30^, ^32^ Also relevant to telerehabilitation interventions with older people, although cognition can have a significant impact on ability to use iPad technology, there is evidence that some people with dementia are able to do so.^29^, ^33^

## Limitations

This study, as for qualitative work in general, is highly context specific. Whilst this has the advantage of providing rich, contextual insight into telerehabilitation experiences, it does pose limitations in terms of the extent to which its insights are applicable to other communities. Previous qualitative work examining patient experiences of telerehabilitation programmes was conducted in north‐western Europe and Canada.^9^, ^14^, ^15^ The alignment in results suggest that the experiences of patients in the present study are similar to those of people in other Western countries. However, a review of health informatics (including telehealth) research in relation to the delivery of care to older people indicates that the majority of studies are conducted in Western countries; thus, there is limited information on cultural factors that may impact on experiences and acceptability of telerehabilitation.^34^ Further research in a range of contexts and cultures will contribute to a broader picture of experiences and acceptability of telerehabilitation to older people.

Although clearly the telerehabilitation technology was acceptable, usable and convenient, it is difficult in this study to isolate the impact of telehealth on therapeutic relationships and service model preferences, as participants' experiences were grounded in both telehealth and in‐person care.

It was planned to include people in this study who declined participation in the larger telerehabilitation trial. This was considered an important design consideration in terms of the generalizability and validity of insights into the acceptability of telerehabilitation, as it would increase the sample size and ensure that decliners were also given a voice. However, all trial decliners also declined participation in this qualitative study, although the study did include insights from ‘converts’ who were initially reluctant to participate but changed their minds. Information from trial refusers can provide important insights into the reasons for non‐participation, which help to better understand the slow adoption of telehealth services.^2^ Qualitative research with telehealth trial decliners has revealed that factors influencing refusal to participate relate to apprehensions about technology, loss of independence and changes to existing services.^2^

## Conclusions

This study provides additional insights to previous research indicating that telerehabilitation is acceptable to older people from Western cultures. Participants in this study perceived telerehabilitation positively, found it convenient, coped well with the iPad and FitBit^R^ technology and developed positive relationships with therapists. The insights from this study highlight some important implications for the on‐going provision of rehabilitation services to older people into the future. Specifically, our results indicate that the expanding use of technology to provide such services at distance is workable and acceptable to older people, and a viable way of translating evidence into practice by increasing exercise dosage. However, there are a number of caveats that should be considered and addressed in the development and establishment of telerehabilitation services. Insights from this and previous research indicate that rehabilitation patients value face‐to‐face contact with their therapist, even when they are very positive about their telerehabilitation experience. This perception of telerehabilitation as complementary rather than a substitute to in‐person care indicates that an ideal telerehabilitation service would continue to provide traditional therapy options by interspersing face‐to‐face contact with at distance therapy wherever possible.

Use of ‘off‐the‐shelf’ technologies, or technologies that are similarly featured, may decrease apprehension and increase usability due to previous exposure through younger family members. Provision of technological support and rapid resolution of technical problems, along with clear instructions, adequate training and support and further, tailored technological developments (such as adjustable stands and high visibility controls) could further increase ease of use and diminish safety risks. Future areas of research that will build on these insights and further inform the development of viable telerehabilitation services include examinations of the conditions under which at distance only telerehabilitation is acceptable, the feasibility of using telehealth technologies to support the rehabilitation of older people with cognitive impairment, and giving a voice to older people who decline telerehabilitation services.

## Sources of funding

This research was supported by a Flinders University, South Australia project ‘*Telehealth in the Home: Aged and Palliative Care in South Australia*’, an initiative funded by the Australian Government.

## Conflict of interests

No conflict of interests to declare.
